# Book Review of Global Medical Physics: A Guide for International Collaboration By Jacob Van Dyk, CRC Press, Boca Raton. 2026. pp. 1–226. ISBN: 978‐1‐032‐86487‐7

**DOI:** 10.1002/acm2.70590

**Published:** 2026-04-19

**Authors:** Matthew Podgorsak

**Affiliations:** ^1^ Department of Radiation Medicine Roswell Park Comprehensive Cancer Center Buffalo New York USA

## 1 OVERVIEW

As stated in its Preface, the intent of this book is to introduce the medical physics community to the need for international collaboration in clinical, educational, academic, and research environments with the aim of improving global health. The editor is Jacob Van Dyk, DSc, a pre‐eminent medical physicist who has been very active throughout his 5‐decade career in promoting the need for global collaboration to improve all aspects of healthcare. Notably, Dr. Van Dyk is a founding member and first president of the Medical Physics for World Benefit (MPWB) organization, whose overall objective, according to the organization's website (mpwb.org) is “To provide medical physics support with the goal of improving the effectiveness and safety in the use of physics and technologies in medicine, especially in low‐to‐middle income countries.”

This book is an excellent resource for everyday medical physicists who would like to become more active in supporting collaborations with colleagues around the world, and I can think of no one better than Dr. Van Dyk to serve as its editor.

## 2 ORGANIZATION

The book is comprised of 18 chapters, each focused on a complementary aspect of global collaboration within the 3 professional environments in which medical physicists work: clinical, educational, and research. In addition to Dr. Van Dyk, there are 34 contributing authors who present their experiences gathered in 21 countries. Each contributor is known for supporting global health initiatives through their own international collaborations. Every chapter begins with learning objectives and ends with a summary and recommendations for applying the chapter's content, along with a comprehensive reference list.

## 3 CHAPTER CONTENTS

The first chapter starts with an introduction to the terminology used throughout the book. Next, the global medical physics workforce is described, focusing on disparities existing among the number of practicing medical physicists, their education and training pathways, and varying recognitions of the medical physics profession throughout the world.

The next two chapters present suggestions for how medical physicists can engage in meaningful collaborations with global colleagues. The need for a well‐defined scope, how the project will unfold, the desired outcomes, and how it will conclude and whether or not there will be any subsequent check‐ins or other follow‐ups are mentioned as key concepts that must be developed for a collaboration to be successful. The launch of AAPM's International Council in 2021 with the associated 3 elements for global collaboration (needs assessment, development of global liaisons, and data and information exchange) is described, leaving the reader with a greater appreciation of the critical details that must be considered when building an inter‐institutional collaboration that includes international collaborators. Significant challenges, such as disparities in available technology, infrastructure, administrative skills, and professional ethics among the collaborating institutions and parties are described as potential roadblocks to a successful project outcome.

Chapter 4 showcases a successful global collaboration initiative: the development of the Radiation Planning Assistant (RPA), a tool that can be used to assist oncologists in low and middle‐income countries to plan radiation therapy treatments. Professionals in Africa and Asia worked with North American collaborators to develop a suite of automated planning tools tuned to the capabilities of the local treatment delivery infrastructure. This product is now in clinical use and is available free of charge to clinics that would otherwise not have access to these planning capabilities. Further description of the RPA collaboration allows the authors to explore the intricacies of a successful collaboration that included research staff, clinical staff, different team sizes, varying timeframes, and different types of organizations.

The benefits of global collaboration with industry are described in Chapter 5 within the context of addressing Africa's cancer crisis. A very detailed discussion on fostering global industry collaborations is presented through description of necessary management approaches along with success metrics. The chapter ends with 4 case studies of collaborations among industry partners and global medical physicists and other clinicians that resulted in highly impactful clinical programs that continue to improve healthcare in Africa.

Chapter 6 describes the sustainability of global collaborations as being dependent on contextual and program factors. Highlighted considerations include the probability for natural disasters, the local political environment, vigor of national commitment, local leadership, financing, project design, and technical expertise, that form the basis for a sustainability framework that must be present for there to be a successful global collaboration. Criteria and situations that may indicate barriers to sustainability are presented.

Chapter 7 focuses on a framework for trainees within any of the existing career paths in medical physics (clinical practice, academia, research, and industry) who are interested in global concerns to structure their studies to align with meeting the needs of global healthcare. The reader is reminded that medical physicists can play a large role in transforming global healthcare, both by sharing technical expertise and through mentorship of global colleagues, as described in Chapter 8.

Necessary components for a global collaboration to be successful along with barriers that must be overcome are addressed in Chapter 9. A list of ‘success’ factors and a comprehensive list of other factors impacting collaboration failure are given. An example highlighting success in global collaboration among clinicians in radiation medicine at different institutions is presented: the launch in 2023 of the IAEA Rays of Hope Anchor Centers. As of December 2025, there are 18 Anchor Centers around the world that work together to elevate their training and research programs.

Chapters 10, 11, and 12 address education and training, advocacy and science diplomacy, and the role of AI, respectively, in global collaborations. Chapter 13 highlights the need for data sharing among global collaborators and describes some existing technologies that facilitate the sharing of data across different jurisdictions that regulate this process. A fascinating exposé of the European Council for Nuclear Research (CERN) experience with global collaborations is given in Chapter 14. Founded in 1954, CERN is a multi‐national organization currently comprised of 24 member states, whose facilities and equipment can be used by any number of member states for research projects. Many important basic science discoveries have resulted from research done by scientists from different countries. The CERN‐Model and its principles are presented as inspiration that can be used when developing the framework for global collaborations.

Chapters 15 to 17 present other important concepts that need to be addressed and properly managed when contemplating a global collaboration. These include ethical considerations, equity, diversity, and inclusion issues, and the pros and cons of different organizational structures found at the collaborators’ institutions.

The last chapter is a brief summary of the entire book. A very nice compilation of the contributing authors’ recommendations in the preceding chapters is given in tabular form and presents a useful framework for increasing the likelihood of success of a global collaboration.

## 4 SUMMARY

This handbook very nicely describes the complexities of a global collaboration. Dr. Van Dyk and the other contributing authors have done an excellent job of presenting content in an engaging and highly informative manner. After reading the book, I found myself to have an increased interest in how global health can be impacted through international collaborations among colleagues who are like‐minded, and I am inspired to contribute to this endeavor. I suspect many readers will feel similarly inspired. I encourage anyone who is interested in supporting initiatives that aim to impact global health to read this highly informative book and to become better acquainted with the AAPM International Council's activities and the objectives of the Medical Physics for World Benefit organization.

